# Successful Repair of Non-facing Sinus ALCAPA Associated With Left-Sided Cardiomegaly Using Takeuchi Technique

**DOI:** 10.7759/cureus.17493

**Published:** 2021-08-27

**Authors:** Vishal V Bhende, Tanishq S Sharma, Hardil P Majmudar, Sohilkhan R Pathan, Deepak V Mehta

**Affiliations:** 1 Pediatric Cardiac Surgery, Bhanubhai and Madhuben Patel Cardiac Centre, Shree Krishna Hospital, Karamsad, IND; 2 Medicine, Shree Krishna Hospital, Karamsad, IND; 3 Pediatrics, Bhanubhai and Madhuben Patel Cardiac Centre, Shree Krishna Hospital, Karamsad, IND; 4 Clinical Research, Bhanubhai and Madhuben Patel Cardiac Centre, Shree Krishna Hospital, Karamsad, IND; 5 Radiology, Shree Krishna Hospital, Karamsad, IND

**Keywords:** congestive heart failure, anomalous origin of the left coronary artery from pulmonary artery (alcapa), cardiovascular ct, pediatric congenital heart disease, takeuchi repair

## Abstract

Anomalous left coronary artery from the pulmonary artery (ALCAPA) is a rare congenital heart disease that may present isolated or may be associated with other cardiac malformations. Most of the patients develop symptoms during infancy but some may remain asymptomatic up to adulthood. Symptoms range from mild distress to severe irritability and feed intolerance. We report a case of a five-month-old male child who presented with congestive heart failure and was diagnosed as a case of ALCAPA with left atrial and left ventricular dilation based on two-dimensional echocardiography and computed tomography (CT) coronary angiogram. Left main coronary artery was shown to be arising from the posteroinferior wall of main pulmonary artery. Various surgical approaches have been suggested in the repair but the Takeuchi technique was preferred owing to its origin from the non-facing sinus of the pulmonary artery and co-existing dilatation of left atria and ventricle. The surgery was uneventful and there were no postoperative complications. A cardiac CT dynamic study was also done on the follow-up visit five months later and no signs of abnormality or complications were reported. Early intervention is necessary to prevent irreversible cardiac complications and early mortality.

## Introduction

Anomalous left coronary artery from the pulmonary artery (ALCAPA) also known as Bland White Garland syndrome is a rare congenital anomaly of the left coronary artery (LCA) with a prevalence of one in 300,000 births and a very high mortality rate of up to 90% if left untreated in the early days of neonatal life [1]. In this, the LCA instead of emerging from the aorta has its origin from the pulmonary artery. In neonates the pulmonary artery pressure is one-third of the systemic pressure; therefore, the blood from the LCA tends to flow into the pulmonary artery. This leads to reduced blood supply to the myocardium and volume overload in the left atria and left ventricle. This phenomenon is popularly known as the coronary steal phenomenon [2]. We report a case of a five-month-old male child who presented with congestive heart failure and was diagnosed as a case of ALCAPA with left atrial and left ventricular dilation.

## Case presentation

**Figure 1:**
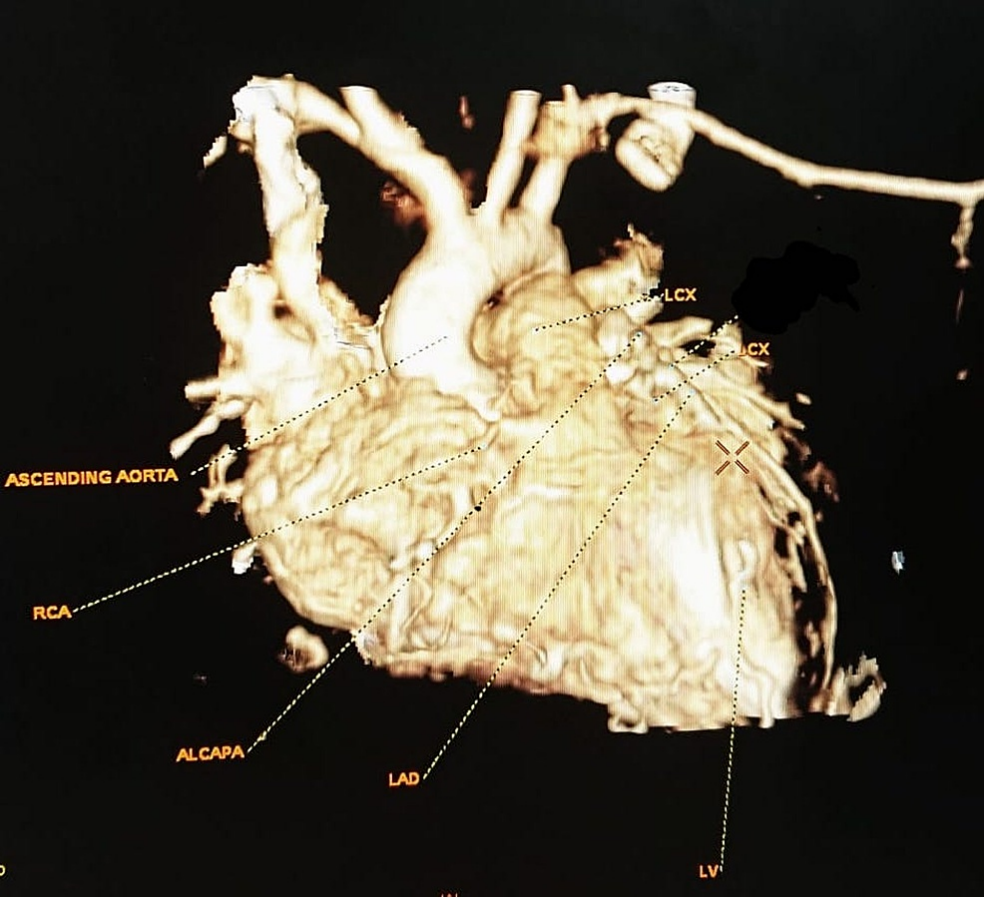
Preoperative Cardiac CT Virtual Reality Scan Showing ALCAPA LCX, left circumflex artery; RCA, right coronary artery; ALCAPA, anomalous left coronary artery from pulmonary artery; LAD, left anterior descending; LV, left ventricle.

A five-month-old male child presented with complaints of irritability and poor feeding. The child also had a history of intermittent episodes of cough and cold. On physical examination, weight for age was 69% indicating grade 2 malnutrition while height for age was 90% indicating grade 1 stunting according to water law’s classification. The heart rate was 140/min, blood pressure was 90/50 mm of Hg, and the patient was tachypneic with a respiratory rate of 62/min. On auscultation bilateral crepitations were audible but no cardiac murmurs were present. A two-dimensional (2D) echocardiography was done, which reported anomalous origin of the LCA from the pulmonary artery, dilatation of left atrium and left ventricle, mild to moderate mitral valve regurgitation, mild pulmonary valve regurgitation, and a patent ductus arteriosus with no evidence of pulmonary artery hypertension. A coronary computed tomography (CT) angiography was done, which showed a cardiothoracic ratio of 58.6% and confirmed the echocardiography findings of ALCAPA. Left main coronary artery was shown to be arising from the posteroinferior wall of main pulmonary artery (MPA) having a diameter of 2.0 mm, and the origin was 15.5 mm distal to the pulmonary valve and 9.0 mm proximal to the bifurcation of MPA. A separate origin of the left circumflex artery from the left coronary sinus was also reported (Figure 1).

**Figure 2:**
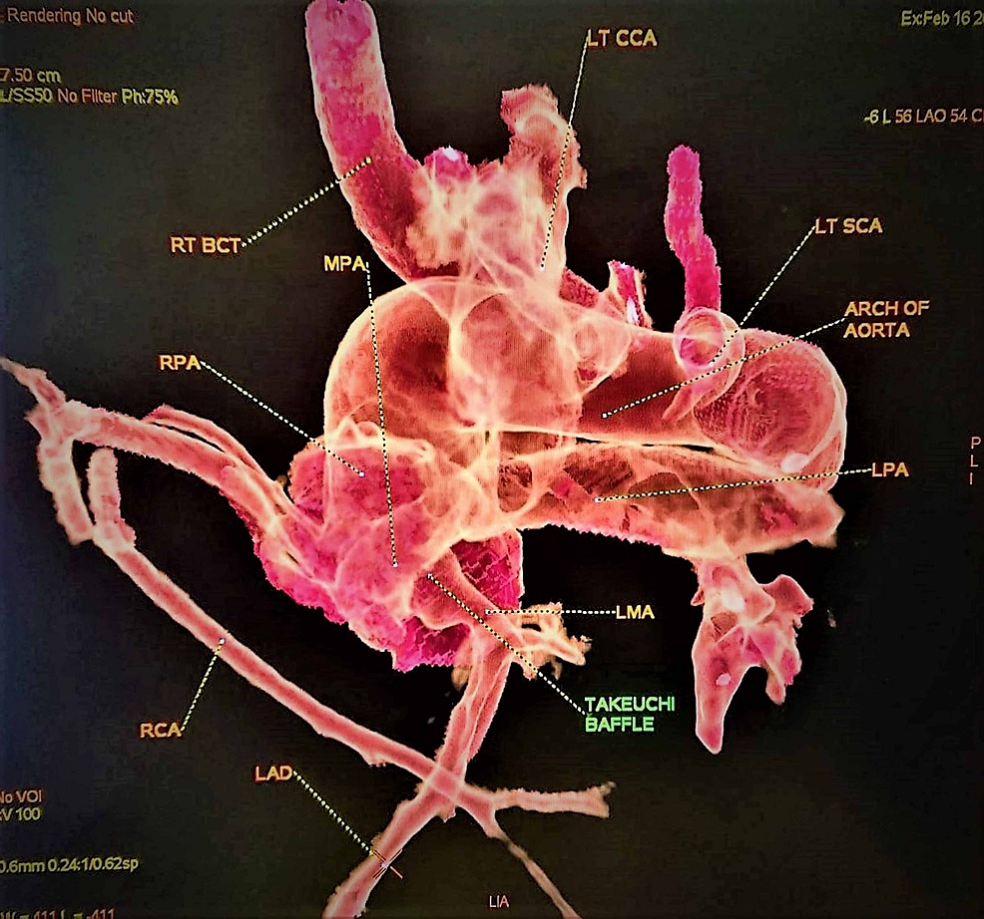
Postoperative Cardiac CT Virtual Reality - Takeuchi Baffle RPA, right pulmonary artery; LPA, left pulmonary artery; RCA, right coronary artery; LAD, left anterior descending; BCT, brachiocephalic trunk; CCA, common carotid artery; SCA, subclavian artery.

He was then shifted to the Cardiac Centre of our hospital and a Takeuchi operation was planned as the LCA orifice was present in the non-facing sinus of the pulmonary artery and there was coexisting dilatation of the left atria and ventricle. The patient was kept on a cardio-pulmonary bypass (heart-lung machine) and a flap of the anterior wall of the pulmonary artery based on its medial wall was created above the sinuses of Valsalva. The anomalous origin of the LCA was visible in the posterior sinus of the Valsalva of the pulmonary artery. A circular opening of about 5.5 mm was created at the base of the flap in the pulmonary artery using an aortic punch. An opening in the medial wall of the aorta was created exactly opposite the pulmonary artery opening using an aortic punch. A side-to-side anastomosis of the aorta to the pulmonary artery was performed to create an aortopulmonary window using continuous stitches of 6-0 prolene sutures. The flap of the anterior wall of the pulmonary artery was displaced posteriorly to cover the back wall of the pulmonary artery and create a passageway within the pulmonary artery from the aorta to the LCA using 7-0 prolene continuous sutures. The anterior wall of the pulmonary artery was augmented with a glutaraldehyde-treated bovine pericardial patch. Direct closure of patent foramen ovale was then done. Chest closure was then done successfully. The patient was kept on a mechanical ventilator till the 18th post-operative day. On the 25th post-operative day, the baby was accepting full feeds and was discharged in stable condition with the advice of regular follow-ups. A cardiac CT dynamic study was done on the follow-up visit five months later and no signs of abnormality or complications regarding the baffle were reported (Figure 2).

## Discussion

ALCAPA has a high mortality rate of 90% if left untreated and contributes about 0.25-0.5% of all congenital heart diseases [1]. Later in 1933, Garland, Bland, and White proposed a review of congenital variations in the coronary vessels and their embryological background. They estimated the incidence of ALCAPA to be 1 in 300,000 live births [3].

Children with congenital heart disease are at a higher risk of suffering from malnutrition, which may lead to growth failure or even failure to thrive. Malnutrition may occur as a result of refusal to feed or reduced tolerance to feed as seen in our patient. Only 10-15% of the untreated patients survive up to adulthood, the reason being the development of large collaterals. Such patients then present with various cardiac complications, which further minimize their survival. About 80-90% of these adults die due to sudden cardiac arrest at the mean age of 35 years [4].

The diagnosis of ALCAPA is very difficult because many patients may remain asymptomatic till the later stages of life. Our patient presented with irritability and refusal to feeds. After 2D echocardiography and CT angiogram, the baby was diagnosed with ALCAPA. ALCAPA mostly occurs as an isolated anomaly of the cardiovascular system but sometimes it may be accompanied by other cardiac deformities. The findings of co-existent mitral regurgitation and dilated cardiomyopathy reported in our case have been mentioned several times in published literature [5].

Management of ALCAPA lies on the restoration of the flow of oxygenated blood through the LCA and reversal of left to right shunt. Various surgical techniques have been described for the repair, which includes ligation of the anomalous artery without reconstruction, saphenous vein or internal mammary artery bypass grafting, Takeuchi repair, coronary re-implantation to the aorta, and anastomosis of the LCA to the subclavian artery. The decision of the best approach depends on the location of the LCA orifice and the presence of coexisting cardiac complications [6,7].

Naimo et al. published a review of 42 children who had undergone surgical repair for ALCAPA. All the surgically managed children at their hospital from the year 1980 to 2014 were included in the study. The survival rate over 10 years was reported to be 86-100% in all the children. Out of the 42 children, the Takeuchi technique was performed at their center in 12 children, and no mortality was noted in this group. Although mortality is low with this technique, complications like supravalvular pulmonary stenosis, baffle obstruction, baffle leaks, and aortic regurgitation can be seen in some patients. In their study out of 12 patients, only one developed stenosis of the MPA 7.5 years later and required a re-operation. Also, after a follow-up of 22 years, none of the patients developed baffle obstruction. Thus, in their study, they concluded that good results can be obtained in patients of ALCAPA by surgery [8].

In our patient, the origin of the LCA orifice was from the non-facing sinus of Valsalva. So direct implantation of the LCA to the aorta was not possible. In such patients, the Takeuchi technique is the best-suggested approach, so we performed the Takeuchi repair and created a baffle at the base of the pulmonary artery from the LCA orifice to the aorta [7].

Hoashi et al. conducted a comparative study between the patients who underwent Takeuchi repair and those who underwent translocation procedure. In their study, the survival rate over 10 years was 87.5% in the Takeuchi group and 71.4% in the translocation group [9].

Ginde et al. in their study have described the exact location of LCA origin in the patients who require Takeuchi repair. On comparing with the existing literature, they found out that the long-term survival rate and freedom from re-operation in the Takeuchi group were similar to that of those who underwent direct implantation [10].

## Conclusions

From the case, we can conclude that the diagnosis of ALCAPA in infants and its early surgical management can prevent further complications and may increase the lifespan of the patient. The Takeuchi technique is a long and complex procedure with risks of multiple early and long-term complications. The technique has to be performed by experienced surgeons to minimize the risks and increase the survival chances of the patient. Many long-term complications have been observed in the patients such as baffle leak and MPA stenosis but they can be managed with re-operation. Therefore, a close and regular follow-up is required for the early detection and management of the complications. Moreover, due to the advancement in imaging techniques, the ALCAPA can now be diagnosed early and easily even in asymptomatic patients. Two-dimensional echocardiography and CT angiogram helped us in the diagnosis of our patient and in deciding the best suitable approach for its management.
